# Plasma creatinine in dogs: intra- and inter-laboratory variation in 10 European veterinary laboratories

**DOI:** 10.1186/1751-0147-53-25

**Published:** 2011-04-10

**Authors:** Tina Ulleberg, Joris Robben, Kathrine M Nordahl, Thomas Ulleberg, Reidun Heiene

**Affiliations:** 1 Ål and Hol Veterinary Clinic, 3570 Ål, Norway; 2 Department of Clinical Sciences of Companion Animals, Faculty of Veterinary Medicine, Universiteit Utrecht, P.O. Box 80154, 3508 TD Utrecht, The Netherlands; 3 Hamar Vetsentrum AS, 2300 Hamar, Norway; 4 If Insurance, POB 240,1366 Lysaker, Norway; 5 Department of Companion Animal Clinical Sciences, Norwegian School of Veterinary Science, PO Box 8146 dep., 0033 Oslo, Norway

## Abstract

**Background:**

There is substantial variation in reported reference intervals for canine plasma creatinine among veterinary laboratories, thereby influencing the clinical assessment of analytical results. The aims of the study was to determine the inter- and intra-laboratory variation in plasma creatinine among 10 veterinary laboratories, and to compare results from each laboratory with the upper limit of its reference interval.

**Methods:**

Samples were collected from 10 healthy dogs, 10 dogs with expected intermediate plasma creatinine concentrations, and 10 dogs with azotemia. Overlap was observed for the first two groups. The 30 samples were divided into 3 batches and shipped in random order by postal delivery for plasma creatinine determination. Statistical testing was performed in accordance with ISO standard methodology.

**Results:**

Inter- and intra-laboratory variation was clinically acceptable as plasma creatinine values for most samples were usually of the same magnitude. A few extreme outliers caused three laboratories to fail statistical testing for consistency. Laboratory sample means above or below the overall sample mean, did not unequivocally reflect high or low reference intervals in that laboratory.

**Conclusions:**

In spite of close analytical results, further standardization among laboratories is warranted. The discrepant reference intervals seem to largely reflect different populations used in establishing the reference intervals, rather than analytical variation due to different laboratory methods.

## Introduction

Creatinine is produced in muscle at a constant rate and excreted by the kidney primarily through glomerular filtration; tubular secretion or reabsorption is negligible. Glomerular filtration rate (GFR) is generally considered the most useful parameter for overall evaluation of renal function and can be estimated by use of urine or plasma clearance methods [1-3]. Clearance methods are time-consuming or require expensive laboratory equipment and are therefore not much used in routine clinical practice, nor in patient screening for research. Plasma creatinine is commonly used as a practical indicator of GFR. A major advantage of plasma creatinine is that laboratory analysis is readily available. The international working group, IRIS (International Renal Interest Society) recommends the use of fasting plasma creatinine as an important clinical parameter for staging chronic kidney disease (CKD) in dogs and cats [4].

Plasma creatinine has limitations with respect to accurately predicting GFR. Firstly, plasma creatinine is a hybrid parameter influenced by both endogenous production in muscle, distribution volume in the body and clearance in the kidney by glomerular filtration. Endogenous production and distribution volumes may vary between individuals. Large dogs have higher plasma creatinine concentrations than small dogs, presumably caused by differences in muscle mass but also clearance, i.e. GFR [1,5-8].

Secondly, analytical error could reduce the usefulness of plasma creatinine as a clinical indicator of GFR. Precision and accuracy of laboratories' analytical results may have an important impact on clinical decision making. A relatively small study in the United States of America indicated that feline plasma creatinine values were, with a few exceptions, similar among laboratories. However, the reference intervals amongst these laboratories were quite different [9].

Standardizing results between laboratories has a high priority in human medicine where creatinine has become more widely used in producing a formula-guided estimated GFR (eGFR). Reference intervals for eGFR take into account gender, race, age, diabetes mellitus status and other clinical parameters [10]. Consensus-based ISO-standards are developed for the purpose of standardization/calibration among laboratories, involving among others the Mandels k- and h-statistic for evaluation of inter- and intra-laboratory variation [11-14]. The adequacy of the statistical methods recommended by the ISO-panels is also under continuous scrutiny [15]. However, broad-scale standardization efforts for creatinine analysis have not been implemented at veterinary laboratories. This lack of standardization among veterinary laboratories may have important clinical implications when interpreting plasma creatinine values, especially values close to the upper limit of the reference interval, which submitting clinicians may consider the cut-off-point between normal and abnormal.

The study was based upon two hypotheses: first, that there is relatively low variation in plasma creatinine values as analyzed by some major veterinary laboratories in Northern Europe; and second, that classification of a sample as normal or abnormal may sometimes reflect differences in reference intervals rather than true differences in analyzed creatinine values.

The aims of this study were thus two-fold: first, to determine inter- and intra laboratory variation as expressed by the statistics of the ISO standard, among 10 veterinary laboratories in Northern Europe, and second, to compare the results from an individual laboratory with its own specific upper reference limit.

## Materials and methods

### Dogs and sample categories

Plasma samples were collected from 30 family-owned dogs in Oslo, Norway and Utrecht, the Netherlands. Inclusion criteria were a body weight above 4 kilograms and age above 1 year. The dogs were categorized according to their expected plasma creatinine values based upon previous laboratory work or a preliminary assessment of the clinical situation. The dogs were divided into three groups: 10 healthy dogs with expected normal values (group 1), 10 dogs with expected intermediate values; i.e. high-normal or mildly azotemic (group 2), and 10 dogs with known azotemia due to kidney disease (group 3).

These pre-defined categories were used throughout the study regardless of the final creatinine concentration measured in the individual dog. After analysis, substantial overlap was observed in the first two groups. The samples were labelled from 1 to 30, with samples from group 1 numbered from 1-10, group 2 numbered 11-20, and group 3 numbered 21-30.

### Sampling and sample handling

The blood samples were collected in heparinised tubes, centrifuged, divided, labelled and frozen in small tubes. Every sample from each dog was divided into 30 aliquots of 0,25 - 0,35 ml plasma. Due to time constraints samples from some dogs had to be frozen before they could be divided into aliquots. These samples were thawed and centrifuged again before preparing the final 30 aliquots. In order to avoid differences in sample handling, thawing and centrifuging was also performed on the samples that had been divided immediately after collection.

Three batches containing aliquots from all 30 dogs were packed frozen and sent by postal mail at 1 to 2 week intervals. The sample and batch number was blinded to the laboratories. Thus, altogether 900 aliquots were shipped in 3 identical batches so that every laboratory received all 30 samples 3 times. The laboratories received the samples by normal postal delivery time, usually 2-4 days, however in some cases up to 7 days. The samples were shipped between March and early May, during which time the temperatures for samples in the mail presumably were similar to the "bench top" temperatures previously studied with respect to stability of analytes in laboratory samples [16].

### Laboratories and analysis of creatinine

The samples were analysed at 10 different veterinary laboratories in 5 countries in Northern Europe, by use of their routine method for creatinine analysis. The utilized laboratories were localized at academic institutions (n = 8) or commercial facilities (n = 2) in Northern Europe, in order to obtain a representative sample of larger laboratories from this part of the world. For reasons of privacy, the participating laboratories are randomly numbered from 1 to 10.

The laboratories were unaware of the exact purpose of the study. However, they were asked to analyse some creatinine samples from dogs with or without kidney disease for a student elective project within the framework of quality control. The laboratories were instructed to analyse the aliquots like any routine plasma sample arriving from any veterinary practice.

We retrospectively sought information about the laboratories methods and reference intervals by sending out a questionnaire to all 10 laboratories. Ten questions were asked about the composition and size of the reference population, statistical methods used for calculation of reference intervals, the type of analytical method and the equipment in use, routines for quality control etc. 9 out of 10 laboratories responded. Two laboratories responded that they did not have access to the data asked for. Among these was laboratory 3 which had the most severely deviating batch in the dataset. The laboratories usually had some, but incomplete, data. Most responders seemed eager to provide the information asked for, but explained missing data by the fact that the reference intervals were created by people different from those currently working in the laboratory, and the original work was not available to them.

Seven out of 9 laboratories provided information on the analytical method in use, among which 4 used enzymatic methods and 3 used the Jaffe reaction. All 7 laboratories used automated large bench top analyzers. All 7 laboratories reported that they routinely performed quality testing against internal and external standards.

Seven out of 9 laboratories provided information on the populations used to create their reference intervals. Only 4 laboratories had information about the size of the reference population. These 4 reference populations consisted of 33, 70, more than 150 or more than 500 dogs, respectively. Three laboratories reported that there was fairly equal gender distribution in the reference population while 4 did not know. All 7 laboratories reported inclusion of many different breeds.

The laboratories' reference intervals have been obtained on fresh samples without prior freezing-thawing, although 2 out of 7 laboratories also have tested the effect of freezing on creatinine analysis without detecting significant discrepancies after freezing and thawing. Five laboratories routinely analyze samples that have been frozen for research projects, using the same reference interval as for unfrozen samples.

Only 1 laboratory reported on the statistical method used for calculation of the reference interval.

### Statistical methods

The International Standard ISO 5725's statistical methods are used to estimate the repeatability, reproducibility and trueness of a standard measurement method [13]. A prototype paper illustrates how the ISO-standard testing can be performed in practical work, and also gives the formulae and explains the statistical parameters used in the current study [14].

Briefly, the basic model in ISO 5725 evaluates the measurement performance of laboratories by 4 different techniques: Mandel's *k *and *h *statistic, Cochran's test and Grubbs test.

The Mandel's *k *statistic and Cochran's *C *statistic are measures of the intra-laboratory consistency. A high *k*-value implies that the laboratory has a high variability when examining the same sample, and in this paper we chose to present the α-significance level of 1%.

The *k*-value is defined as:  where *S*_*i *_is the standard deviation of the laboratory's measurements over one sample, and *S*_*r *_is the estimated repeatability standard deviation.

The C statistic is defined as:  where  is the largest variance from a laboratory within one sample and p is the number of laboratories.

Several outliers within one laboratory in the *k *statistic or Cochran's test are a strong indication that the laboratory has a high intra-laboratory variance. All data from this laboratory may be rejected, if the purpose of the testing is to achieve equivalent/interchangeable laboratory results based upon the ISO standard.

Mandel's *h *statistic and Grubbs G statistic is primarily a measure of the inter-laboratory consistency. The *h*-value is defined as:  In this formula  is the average measured result of one sample from a specific laboratory,  is the mean result of all laboratories and *S*_*m *_is the corresponding estimated standard deviation. If the *h*-value for one laboratory is significantly above or below zero, this implies that the laboratory provides a biased result, and in this paper we chose to present the α-significance level of 1%.

Grubb's test statistic *G*_*k *_is defined by:  In this formula *y*_*k *_is one measured result,  is the average of all the observations and s is the corresponding estimated standard deviation. The test could either be used on all measured results to reveal outliers from a normal distribution, or alternatively, if used to test individual average laboratory results, the formula becomes quite similar to Mandel's *h*.

Outliers among laboratories in the *h *statistic or Grubb's test provide a strong indication that the laboratory caused a high inter-laboratory variance. This laboratory may in such cases be rejected, if the purpose of the testing is to achieve equivalent/interchangeable laboratory results based upon the ISO standard.

Outliers detected by these 4 statistical tests were not removed from our dataset, in accordance with our aim of comparing all results from the 10 veterinary laboratories. Thus, the deviating results were allowed to create a bias.

One exception was made in figure four where we did remove the extreme outlying values from 2 batches in laboratory 3 (batch 3) and 4 (batch 1), which created a lot of bias throughout all tests. Such extreme outliers would normally be removed from a research dataset. In figure four the objective was not to test the laboratories but to evaluate the measured values relative to the reference intervals. Thus the figure is considered more illustrative when the outliers are removed. SD in percent of the mean value (from each batch, illustrating variation between laboratories) was calculated after removing the same batches.

Additionally, Pearson's correlation coefficient was calculated between the laboratories' mean analytical result in all groups and the upper limits of their reported reference interval.

All statistical testing was performed on log-transformed data in Office Excel (Microsoft; Mountain View, CA).

## Results

Laboratory 7 had to freeze all the samples from the first batch and analysed this batch in the same period as the second batch. Statistical analysis demonstrated no deviation in the laboratory results of these samples in spite of the one extra freezing/thawing step, and they were therefore accepted for further analysis in the dataset. The same laboratory also reported that the third batch contained an insufficient volume in some of the samples. However, they were still able to analyze them and obtained non-deviant results. None of the other laboratories reported technical problems.

**Figure 1:**
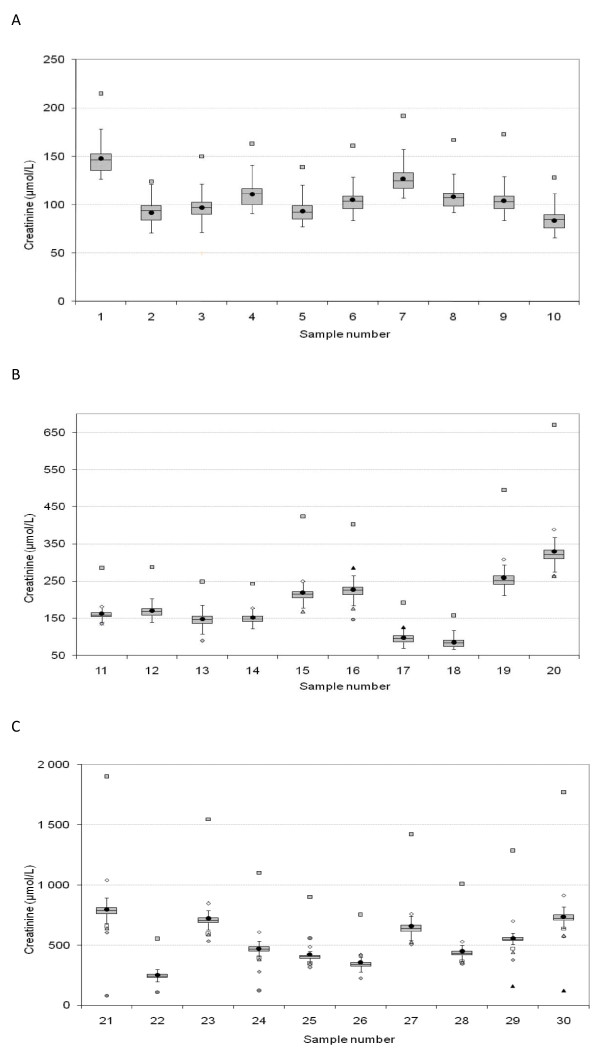
**Box-Jenkin plots of plasma creatinine values in plasma samples from A: 10 healthy dogs; group 1 (Sample no 1-10) and B: 10 dogs with expected intermediate values; group 2 (Sample no 11-20) and C: 10 azotemic dogs; group 3 (Sample no 21-30) analyzed 3 times in different batches by 10 laboratories in Northern Europe**. The box represents the interquartile range from the 25^th ^to the 75^th ^percentile. The horizontal bar through the box is the median. The whiskers represent the main body of the data; that is, the upper or lower quartile ± 1,5× interquartile distance. Outlier values (outside the 1,5× interquartile distance) are represented by different symbols for each laboratory; grey squares represent one outlier batch from laboratory 3.

Analytical values showed small variation between laboratories and from batch to batch with exception of a few extreme outlier values - particularly batch 3 from laboratory 3, as can be appreciated in Table 1 and Figure 1. Standard deviation (%) for each of the 3 batches in all 10 laboratories was average 9,6%. The variation was larger amongst the dogs with azotemia (group 3) than the dogs with lower plasma creatinine values (groups 1 and 2).

**Table 1:** Raw data

		Sample number																													
Laboratory		*1*	*2*	*3*	*4*	*5*	*6*	*7*	*8*	*9*	*10*	*11*	*12*	*13*	*14*	*15*	*16*	*17*	*18*	*19*	*20*	*21*	*22*	*23*	*24*	*25*	*26*	*27*	*28*	*29*	*30*
***Lab 1***	batch 1	151	95	96	111	95	103	130	109	104	88	159	170	142	144	207	214	94	87	247	320	762	247	692	452	400	314	619	418	542	715
	batch 2	147	93	93	107	93	99	127	106	102	85	156	168	141	139	202	146	77	84	243	309	754	243	688	450	396	309	616	415	539	709
	batch 3	151	96	99	110	98	104	133	112	106	90	161	173	143	145	211	214	100	86	248	322	766	247	689	454	400	314	619	426	544	715

***Lab 2***	batch 1	153	95	107	121	101	107	137	111	122	91	171	175	160	161	222	237	112	93	269	305	816	261	731	488	398	334	664	448	567	749
	batch 2	144	98	95	113	89	104	125	105	102	90	164	171	155	152	219	227	102	81	251	333	783	255	699	468	405	319	618	430	577	726
	batch 3	152	91	100	109	92	105	125	108	108	87	158	167	156	154	221	219	76	84	254	335	790	236	707	478	407	313	620	449	551	728

***Lab 3***	batch 1	149	84	85	103	79	93	126	102	97	78	162	168	146	146	215	215	88	83	250	333	860	272	723	507	415	355	650	457	550	709
	batch 2	174	98	103	119	98	120	144	119	113	87	181	195	170	177	250	250	100	96	308	389	1042	293	851	611	488	421	763	531	703	915
	batch 3	**215**	**124**	**150**	**163**	**139**	**161**	**192**	**167**	**173**	**128**	**285**	**287**	**249**	**243**	**424**	**403**	**192**	**157**	**495**	**671**	**1905**	**555**	**1546**	**1103**	**900**	**757**	**1420**	**1010**	**1289**	**1774**

***Lab 4***	batch 1	145	104	98	137	97	125	133	110	109	84	156	181	151	165	224	285	125	93	271	348	859	270	743	502	408	370	675	495	**163**	**123**
	batch 2	142	96	85	112	99	90	118	107	105	77	160	178	140	154	195	230	107	85	227	331	**82**	**110**	715	**124**	560	346	649	466	546	738
	batch 3	130	71	**50**	91	77	102	107	107	90	66	157	154	139	155	204	234	87	70	257	320	856	254	716	498	408	348	655	484	563	756

***Lab 5***	batch 1	127	84	91	100	83	98	111	95	92	75	135	142	**89**	127	181	188	76	75	222	262	606	206	535	281	317	226	506	342	376	574
	batch 2	132	96	105	108	91	110	117	98	100	83	147	155	152	142	188	204	100	80	235	276	659	218	597	396	350	387	568	365	472	639
	batch 3	127	85	102	100	88	96	115	96	96	78	141	138	131	121	167	175	86	73	212	262	643	212	591	383	347	422	528	358	442	581

***Lab 6***	batch 1	134	78	84	96	79	96	114	92	88	70	147	161	135	135	214	225	71	67	248	317	791	237	707	458	406	348	643	418	542	757
	batch 2	140	77	91	100	79	105	111	99	96	67	154	164	134	141	221	240	88	70	256	313	789	235	742	468	408	355	669	426	574	756
	batch 3	129	82	84	95	82	89	120	95	84	69	158	157	131	148	221	234	69	70	240	312	829	233	706	462	398	351	670	421	553	760

***Lab 7***	batch 1	135	75	95	94	104	95	113	93	90	68	135	151	120	138	204	202	94	69	240	305	800	238	677	480	383	334	615	432	542	719
	batch 2	137	74	87	91	93	89	113	97	90	68	145	153	122	141	198	199	94	69	236	308	804	235	688	472	383	337	618	439	545	730
	batch 3	134	74	86	93	88	92	118	95	89	68	145	151	127	140	201	197	98	70	246	318	809	235	673	472	385	337	609	431	545	731

***Lab 8***	batch 1	159	103	109	117	105	122	140	119	109	96	166	165	149	146	207	222	88	94	242	314	721	247	667	435	386	325	592	407	515	666
	batch 2	162	112	118	126	113	126	147	126	118	103	177	186	159	160	253	237	94	97	261	333	776	261	697	457	409	344	630	427	547	710
	batch 3	154	104	104	114	102	114	131	116	113	95	165	178	145	148	205	218	100	91	245	309	718	247	663	432	388	326	598	412	528	676

***Lab 9***	batch 1	148	91	98	113	90	106	128	107	106	86	156	168	141	148	214	223	104	84	256	320	786	246	707	457	403	348	644	416	555	724
	batch 2	158	100	105	119	100	84	133	128	107	91	163	176	149	158	218	228	89	89	265	335	813	266	739	488	433	365	669	449	579	754
	batch 3	160	99	101	115	99	113	137	114	110	92	170	178	151	161	230	230	103	91	266	341	835	271	752	496	440	382	680	461	592	777

***Lab 10***	batch 1	151	100	100	125	88	105	125	107	113	87	169	174	170	151	227	254	117	87	274	324	796	263	726	477	417	328	680	434	558	754
	batch 2	146	89	90	112	83	98	117	101	97	79	157	160	164	141	222	230	77	80	262	314	780	250	711	460	412	317	636	416	540	727
	batch 3	146	91	96	114	84	101	122	106	98	82	165	167	163	147	225	228	78	84	263	325	807	253	729	471	421	331	657	425	565	750

Results from the dogs with expected normal values are labelled 1-10, dogs with expected intermediate values 11-20, and dogs with known azotemia 21-30. Observations highlighted are more than 2 standard deviations from the empirical mean

**Figure 2:**
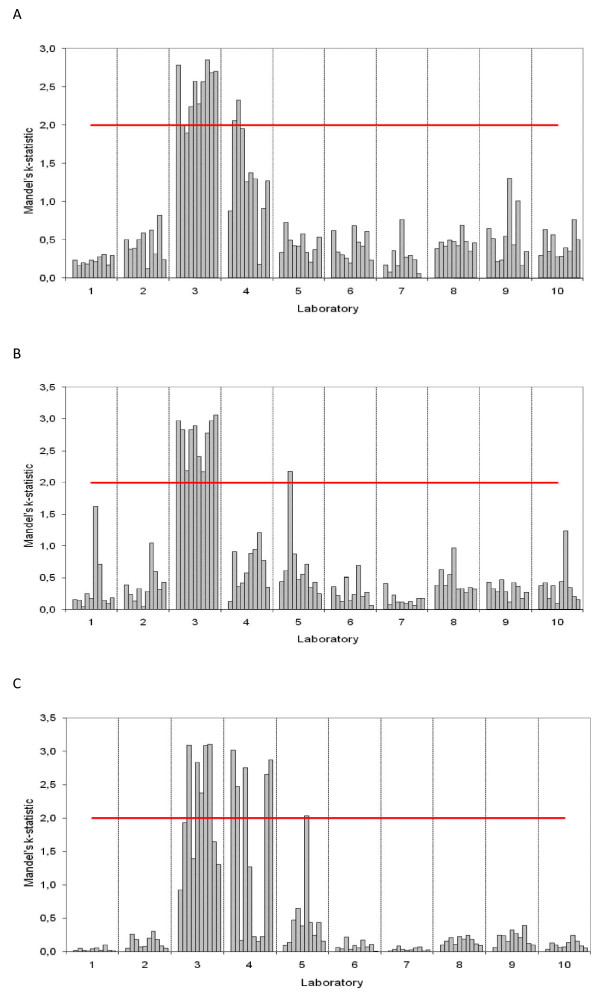
**Mandels k-statistic plots, illustrating intra-laboratory variability in analytical results for plasma creatinine for each of 10 European laboratories (1-10)**. Every bar represents the k-value of one sample that has been analyzed 3 times in different batches. A high k-value indicates a high intra-laboratory variance. The horizontal line defines the 1% α-significance level. A: 10 healthy dogs, B: 10 dogs with expected intermediate values and C: 10 azotemic dogs.

The Mandels *k*-statistic (Figure 2) illustrates how the outliers in laboratory 3 and 4 substantially increased the intra-laboratory variation. The batches from laboratory 3, due to one specific deviating batch, in addition to some batches from laboratory 4, failed the Cochrans' test for intra-laboratory consistency. From that specific batch in laboratory 3, analytical results were of approximately double magnitude to the analytical result in the other two batches for most of the 30 samples (Table 1).

In all groups and samples the deviating batch from laboratory 3 was a major contributor to the differences observed.

**Figure 3:**
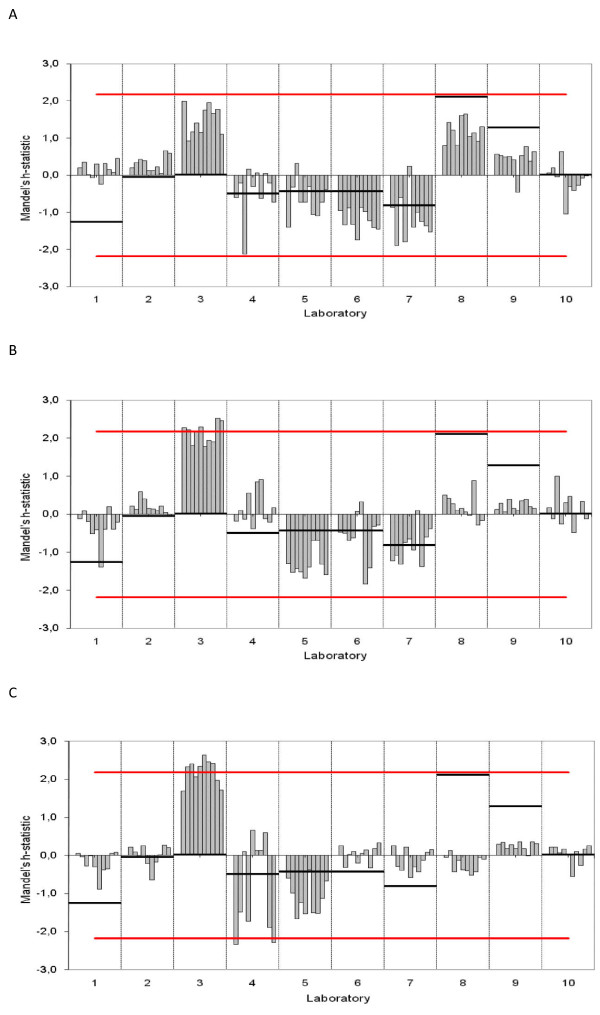
**Mandels h-statistic plots, illustrating inter-laboratory variability in analytical results for plasma creatinine for each of 10 European laboratories (1-10)**. Every bar represents the h-value of one sample that has been analyzed 3 times in different batches. A high h-value indicates a high inter-laboratory variance. The short horizontal lines indicate the distance, in standard deviations, of the upper reference limit of the individual laboratory from the mean of all laboratories' upper reference limit. The horizontal line defines the 1% α-significance level. A: 10 healthy dogs, B: 10 dogs with expected intermediate values and C: 10 azotemic dogs.

According to the Mandels *h*-statistic (Figure 3) laboratory 3 and 8 provided analytical results above the other laboratories, that is, a positive bias (bar above 0), while laboratory 5, 6 and 7 are negatively biased in this study. Laboratory 4 is negatively biased in healthy and azotemic dogs, but positively biased in dogs with expected intermediate values. Figure 3 illustrates that a specific laboratory's upper reference limit relative to the mean upper reference limit of all laboratories, is not always related to the magnitude or direction of the bias in the results from that laboratory. While laboratories 3 and 4 had deviations outside the 1% significance level in intermediate or azotemic dogs, no laboratories had deviations outside the 1% significance level among healthy dogs.

Extreme individual outliers from a normal distribution, based on the Grubbs test, were as follows: In laboratory 3, 16 out of 30 outlier samples were in the third batch. In laboratory 4, two outlier samples were in the first batch, three samples in the second batch and one sample in the third batch.

Laboratory 3, due to deviating samples, failed Grubbs test for inter-laboratory consistency on the 1% α-significance level.

The correlation coefficient between mean analytical results and the upper limits of the reference intervals of the 10 laboratories were 0,60 for group 1; 0,31 for group 2 and 0,15 for group 3, respectively.

**Figure 4:**
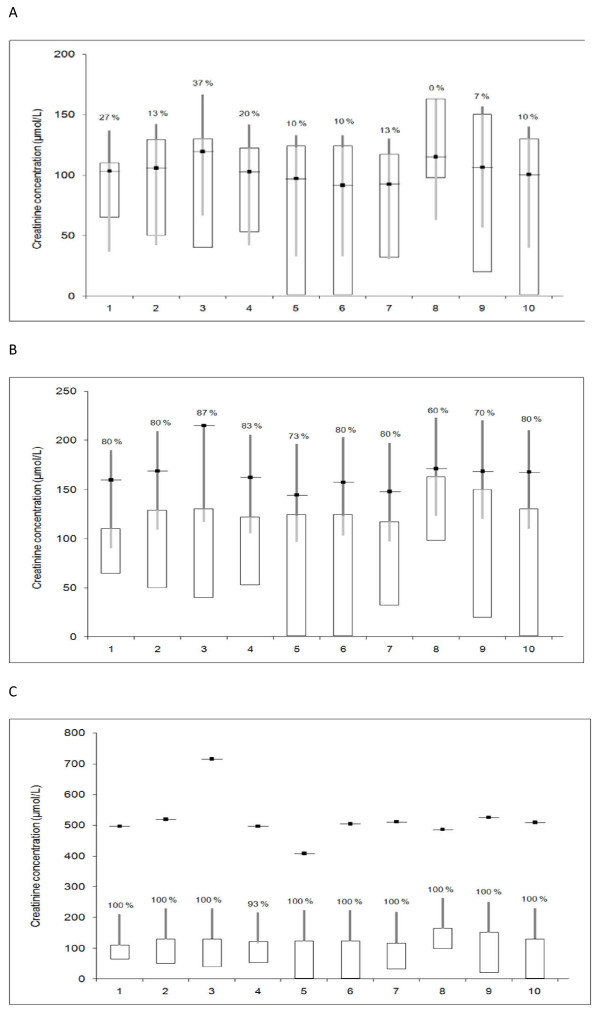
**Median creatinine concentrations (μmol/L) and ranges in 10 healthy dogs, 10 dogs with expected intermediate creatinine values and 10 azotemic dogs, analyzed 3 times in different batches by 10 European laboratories (1-10)**. The horizontal line represent the median. The white background box indicates the reference interval in healthy dogs as specified by each laboratory. Dark grey columns overlaid the upper reference limit, and figures given above them, represent the percentage of dogs that are considered abnormal by use of the upper reference limit for that laboratory. The light grey columns represent the percentage of dogs that are classified as normal. A: 10 healthy dogs, B: 10 dogs with expected intermediate values and C: 10 azotemic dogs.

The median values in groups 1, 2 and 3 are plotted for each of the individual laboratories, with their respective reference intervals included, in Figure 4a,b and 4c.

In Figure 4a and 4b, the data is plotted for the two groups where a substantial number of samples in each group were classified as normal or abnormal. In the laboratory with the lowest and highest reference interval (laboratory 1 and 8), 27% and 0% of the healthy dogs were classified as abnormal, respectively. When comparing percentages of healthy dogs classified as abnormal in laboratory 4 and 5, there is a clear difference in spite of nearly identical upper reference limits. Also, when comparing percentages classified as abnormal in laboratory 2 and 3, there is a clear difference in spite of the similar upper reference limits.

## Discussion

This study indicates that while most analytical results are of the same magnitude in the laboratories examined, the classification of the sample as normal or abnormal may differ due to the variation in reference intervals specified by each laboratory. In addition, a few outlier values represent a problem.

To be valid, a laboratory's reference interval should account for differences in chemical methodology and reproducibility of that method, but statistical methodological concerns are also of importance. The statistical prerequisites for a valid reference interval includes 1) that the sampled population is representative for the population from which the laboratory will receive samples, 2) that the number of samples is large enough, and 3) that the statistical methods used for calculation of the reference interval are valid. In practice, there are problems with all 3 prerequisites in veterinary laboratories. Regarding 1) there is breed, size, gender and age variation in plasma creatinine, and a reference population would have to be extremely large to be truly representative. Regarding 2) the sample size used for calculation of reference limits is often small, exemplified by 33 dogs in one of the laboratories used in this study. Regarding 3), the underlying distribution must be known in order to use the correct methods for value distributions in a population, which may be difficult with a small number of animals.

A smaller study with overlapping aims with this study, is presented as an abstract from US laboratories [9]. Also in that study, classification of a sample differed from laboratory to laboratory due to variation in reference intervals, in spite of analytical results of the same magnitude for plasma creatinine in many samples.

Plasma creatinine is routinely used to screen for kidney disease and a rational approach to the clinical use of this diagnostic test is important. A clinician or researcher will commonly consider the laboratory's upper reference interval as the cut-off value to distinguish normal from abnormal. The optimal cut off point for a simple test against a golden standard is sometimes evaluated by use of receiver operating curve (ROC) analysis, aiming at a cut off point with high sensitivity and specificity. Based upon GFR as estimated by exogenous creatinine clearance, one recent study in 232 dogs defined the optimal cut off value for plasma creatinine to be 144 μmol/L [17]. This value is intermediate relative to the upper reference intervals of the 10 laboratories in this study. That study illustrates the difference between reference intervals for a laboratory and decision thresholds for individual patients.

While there are different ways to perform a comparison of laboratories' analytical results, in this study we made use of consensus-based statistical methods from international ISO-panels, as detailed in ISO-standard No 5725 [13]. When applying the ISO-standard for calibration of laboratory values, some of the laboratories and batches in this study failed the statistical testing. The results from laboratory 3 are severely biased due to one specific highly deviating batch. Generally, a clinician will recommend repeated testing if unexpected laboratory results appear. However, for the dogs in this study, the outlier result could have been accepted as consistent with the clinical situation. This illustrates the need for veterinary laboratories to focus on calibration and consistency amongst their results.

In human clinical pathology laboratories, creatinine standards in human plasma with known creatinine content are available. It may be premature to apply the stringent ISO standard developed for human medicine in veterinary medicine. Quality control efforts in veterinary laboratories have often observed large discrepancies across methodologies for analysis of proteins, which may be expected for creatinine. Thus for most proteins results are only comparable within methodology, and establishment of local reference limits are of great importance.

However, the inter-laboratory variation across methodologies in the 10 laboratories in this study may be considered low in this context, as illustrated by the fact that only the deviating batches from laboratory 3 and 4 created differences in analytical results of clinical significance (as illustrated in Table 1, Figure 2 and Figure 4).

The laboratories with the highest or lowest reference intervals did not unequivocally produce values that were correspondingly low or high relative to the overall mean analytical result (Figure 4). For instance, laboratory 8, 9 and 10 did produce very similar results, while their upper reference limits are quite different. Laboratory 1 gives the lowest upper reference limit, but the measured plasma creatinine in the 10 healthy dogs in laboratory 1 was not lower than in laboratory 5, 6 and 7, which provide higher upper reference limits. This illustrates how the laboratories' upper reference limits do not satisfactorily "calibrate" the measured result to the laboratory's analytical method. To the contrary, for some dogs there is an element of arbitrary classification of the patient as normal or abnormal depending upon which laboratory was used.

The results show a positive correlation between the upper reference limits and the analytical results for the laboratories. However, because the upper reference limit defines normal or abnormal results from that laboratory, one would expect a strong correlation between the reference interval and the analytical result in a laboratory. In this context, the observed correlation is not very strong.

The variation in plasma creatinine due to age, gender and breed is likely much greater in dogs where the sizes may vary from 1,5 to 80 kg in adult dogs, and both clearance and plasma creatinine may vary in different sizes and breeds [1,18]. In one study including several hundred dogs, the upper reference limit (mean+2SD) during interim analysis was 90 μmol/L for dogs < 10 kg and 178 μmol/L for dogs above 45 kg; which corresponds to the creatinine concentration detected in another large study where a large number of small and large dogs was included [18]. Very similar results were found in a dataset of several hundred dogs in our institution[5]. Thus, it is not unexpected if the composition of the reference population used in a laboratory substantially influences the results for plasma creatinine.

The summarized acceptable error (random and systematic) that can be tolerated in laboratory analysis is termed total allowable error. This is an important measure for any laboratory analysis. However, until the above mentioned studies are published [5,18], it is difficult to quantify total allowable error for plasma creatinine in veterinary medicine. A veterinarian needs knowledge about various biological causes for variation that add to the laboratory variation. The decision thresholds for considering a dog abnormal does not directly correspond to the reference interval of the laboratories. Given the observed values of up to double magnitude in giant dogs compared to miniature dogs, one may question the usefulness of a single upper reference limit for creatinine in dogs. Accuracy in laboratory analysis nevertheless remains a prerequisite for evaluation of patients.

The population used by each laboratory for defining its reference intervals differ in terms of age, breed and body size. This may cause discrepant reference intervals in different laboratories. Given the relatively small variation in analytical results in the groups of healthy dogs and dogs with intermediate values (Figure 1a and 1b; Table 1), the fairly large discrepancies between the proportion of dogs classified as normal or abnormal when analysed in different laboratories (Figure 4a and 4b) are striking to a clinician. The findings in this study thus support the hypothesis the differences in upper reference limits result from non-uniform reference populations used by the laboratories, rather than a bias based primarily upon the analytical method as such.

The IRIS staging system for CKD is based upon fasting plasma creatinine, with sub-staging based on urine protein/creatinine ratio and systolic blood pressure. The IRIS classification system makes use of the actual measured plasma creatinine value without reference to which laboratory is used, nor to the animals' age or body size. The IRIS staging system thereby represents a tool for veterinarians to communicate about patients without depending on the cut off-values for healthy dogs set by any particular laboratory.

The results of the present study support the evaluation of plasma creatinine with some independence relative to the reference limits given by the specific laboratory used. Hence, our results support the use of the IRIS guidelines for classification of chronic kidney disease, particularly for IRIS stage 3 and 4 and probably 2. It is questionable as far as the upper limit of stage 1, where the difference between reference intervals and decision threshold deserves attention. This should be elucidated in future research. The IRIS guidelines apply for average-sized dogs, and some data indicate that the creatinine concentrations are different in very large or very small dogs. ^a ^This further emphasizes that a laboratory should strive to achieve representativity in dog populations used for establishment of reference intervals.

Importantly, methodological differences may be of greater importance if small bench-top analysers are used. It is well known that small bench top (point-of care) laboratory analyzers used by small clinics have very different reference intervals. Such bench top analyzers were examined in a recent study from France, where the analytical results showed great variation from laboratory to laboratory [19]. The analyses were performed in 99 veterinary practices, most often by VetTest (Idexx) and Reflovet (Scil Animal Care). Thus, the conclusions from this study are not valid in a setting where bench top small analyzers are used for measurement plasma creatinine.

There are several limitations to this study. Time for postal delivery could represent a source of error. One batch had been sent out too close to a holiday weekend and thereby spent 7 days in the mail before analysis. Ideally, stability of the samples should have been verified [16,20]. However, the results from the deviant samples were not amongst the batches with the longest mail time. Therefore, we do not consider the time for postal delivery likely to have influenced the results in this study. The duplicate freezing and de-thawing of all samples represent potential sources of error, though the equal treatment of all samples should minimize the influence upon the differences found between laboratories. The laboratory internal reference intervals are obtained on fresh samples without prior freezing-thawing, 2 of the laboratories also have tested the effect of freezing on creatinine ananlysis withouht detecting significant discrepancies after freezing and thawing. Non-creatinine chromogens in a sample can produce error in the measured creatinine value. The most deviating samples in this dataset without exception came from single batches in laboratories where the other 2 of 3 aliquots from the same sample usually produced non-deviating results in the same laboratory. Thus, non-creatinine chromogens likely do not influence the deviating results in this study. Another limitation of the study is the lack of information about the basis for each laboratory's determination of their reported reference interval. This could be defined in future studies where an improved standardization across laboratories is aimed at.

A comparison of the ISO-standard and other statistical approaches to evaluate inter- and intra laboratory variation may be an aim of future research. Establishment of valid reference intervals from more representative dog populations could be undertaken as a multi-center study, where the composition of a representative dog population is defined, and then sample collection is undertaken in a manner that makes the samples available to many laboratories for establishment of local reference intervals.

## Conclusion

The differences in reference intervals among laboratories seem to potentially reflect differences amongst the healthy dog populations that were used in establishing the reference intervals, rather than the applied assay methods only. This study demonstrates a need for standardization efforts in veterinary laboratories, and supports the use of the IRIS staging system when communicating about patients.

## Competing interests

The authors declare that they have no competing interests.

## Authors' contributions

The study was designed by RH, JR, TU and KMN. TU and KMN carried out the study. ThU performed the statistical analysis and created the Figures. RH drafted the manuscript; JR participated in the writing. All authors read, revised and approved the final manuscript.
